# Diseases of the nERVous system: retrotransposon activity in neurodegenerative disease

**DOI:** 10.1186/s13100-019-0176-1

**Published:** 2019-07-26

**Authors:** Oliver H. Tam, Lyle W. Ostrow, Molly Gale Hammell

**Affiliations:** 1 Quantitative Biology, Cold Spring Harbor Laboratory, Cold Spring Harbor, NY 11724 USA; 2 Department of Neurology, Johns Hopkins University School of Medicine, Baltimore, MD 21205 USA

**Keywords:** Transposable elements, Endogenous retroviruses, Neurodegenerative disease, Amyotrophic lateral sclerosis, Alzheimer’s disease, Aicardi-Goutieres syndrome, Multiple sclerosis

## Abstract

Transposable Elements (TEs) are mobile genetic elements whose sequences constitute nearly half of the human genome. Each TE copy can be present in hundreds to thousands of locations within the genome, complicating the genetic and genomic studies of these highly repetitive sequences. The recent development of better tools for evaluating TE derived sequences in genomic studies has enabled an increasing appreciation for the contribution of TEs to human development and disease. While some TEs have contributed novel and beneficial host functions, this review will summarize the evidence for detrimental TE activity in neurodegenerative disorders. Much of the evidence for pathogenicity implicates endogenous retroviruses (ERVs), a subset of TEs that entered the genome by retroviral infections of germline cells in our evolutionary ancestors and have since been passed down as a substantial fraction of the human genome. Human specific ERVs (HERVs) represent some of the youngest ERVs in the genome, and thus are presumed to retain greater function and resultant pathogenic potential.

## Background

Transposable elements (TEs) represent a vast array of genomic sequences that have (or once had) the ability to mobilize from one location in the genome to another. Several excellent reviews explain the general features and behavior of transposable elements [1–3]. Two major classes of TEs exist: Class I TEs, also called retrotransposons, utilize an RNA intermediate that is reverse transcribed before genomic reinsertion; Class II TEs, or DNA transposons, move via excision from one genomic location and insertion into another. In most genomes, Class I retrotransposons represent the vast majority of TE derived sequences since new copies accumulate with each transposition event. Retrotransposons can further be subdivided into a few major families: the long interspersed nuclear element (LINE) class of fully autonomous retroelements (21% of the human genome [4] the SINE class of shorter retroelements that rely on LINE encoded proteins for mobilization (13% [4]), and the long terminal repeat (LTR) retrotransposons that include endogenous retroviruses (ERV, 8%) [4]. ERV sequences [1–3, 5] represent an interesting subclass of TEs that derive from retroviral infections of germline cells in our ancestors, which were then endogenized and passed along to future generations. Some of the evolutionarily youngest (ie, most recently inserted) TEs are present only in the genomes of humans and closely related primates, and are dubbed *human ERVs* (HERVs).

While nearly half of the human genome is composed of TE derived sequences [4], almost all of these sequences have lost the ability to mobilize to new locations. Only the human specific LINE-1 (L1) element, L1HS, present in full form in 100–180 locations in the human genome [6–9], retains the ability to autonomously mobilize and create new insertional mutations. Other TEs have less autonomous levels of function, varying from the simple ability to be transcribed into RNAs, the ability to make functional proteins, the ability to reverse transcribe their RNA transcripts into cDNA, and, finally, the ability to attempt genomic insertion. Thus, while many studies of TEs focus on detection of new transposition events (hopping), their novel functional activities can be broadly classified based on whether they engender RNA expression, cDNA generation, and/or production of functional proteins. Each TE insertion encodes for a different level of activity due to mutations within the TE sequence that may render protein or regulatory sequences non-functional. Thus it is important to be specific about the relative ability for each TE copy to affect cellular function, following the detection of aberrant TE accumulation.

### Mechanisms for TE-mediated cellular stress

The most commonly implicated pathogenic functions of TEs result from direct mutagenic effects of newly transposed insertions. As stated above, only a subset of L1HS elements are fully capable of mobilizing in vivo, creating de novo insertional mutations at a rate of about one L1HS germline insertion per 100 individuals [10]. In addition, L1HS machinery can facilitate mobilization of other non-autonomous TE families, including Alu and SVA (SINE/VNTR/Alu), some of which are known to be polymorphic (representing relatively recent insertion events) with estimated transposition rates of about 0.04 and 0.001 new insertions per generation, respectively [10], and an overall retrotransposition rate of about 0.02 germline events per generation. L1HS can also mobilize in certain somatic tissues, with a transposition rate estimated at about 0.04–13 insertions per cell in neurons [11–15]. This cell-type-specific mosaicism could explain reports suggesting that de novo transposon insertions are more commonly found in brain compared to other somatic tissues [11, 16, 17] and that neuronal cells are more permissive to retrotransposition [5, 11]. However, a comprehensive study comparing somatic transposition rates across healthy human tissues has not been completed. In contrast, somatic retrotransposition is much more common in human cancers [18] with an estimated rate of 4–100 de novo insertions per tumor in many tumor types of different tissues [8, 19, 20]. The potential for similarly higher somatic rates has not been fully explored in disease settings outside of cancer.

In contrast to the relatively rare events of detectable de novo insertion, the most common molecular function of TEs is to generate RNAs. Many genomic TEs retain transcriptional regulatory sequences that can direct the generation of RNA transcripts, potentially including chimeric sequences downstream of the TEs themselves [21–23]. A subset of ERVs and L1 elements contain bidirectional promoters [21] capable of generating long double-stranded RNAs (dsRNAs) [24]. Moreover, the density of sense and anti-sense copies of TEs embedded within introns and untranslated regions creates the potential to generate dsRNA segments from adjacent inverted repeats (IR), with Alu elements being the most common source of IR derived dsRNAs [25, 26]. When not properly degraded or resolved by adenosine deaminase, RNA specific (ADAR) editing complexes, long dsRNAs from retroelements can be recognized by DExD/H-Box Helicase 58 (DDX58/RIG-I), which alerts the antiviral surveillance machinery and activates inflammatory responses via interferon mediated Toll-Like Receptor pathways and tumor necrosis factor (TNF) alpha [24]. In addition, if the TE RNAs are produced from a locus that encodes a functional reverse transcriptase, there exists the possibility for cytosolic cDNA production, which alerts a separate interferon-mediated inflammatory pathway downstream of cyclic GMP-AMP synthase (CGAS) and stimulator of interferon genes (STING/TMEM173) [24], as has previously been seen in aging mice expressing active LINE1 retrotransposons [27, 28]. Finally, some of the proteins generated from full length TE transcripts are directly 1) immunogenic in certain cancers [29, 30], and 2) cytotoxic in the case of HERV derived Envelope proteins in neurodegenerative diseases [31, 32].

**Fig. 1:**
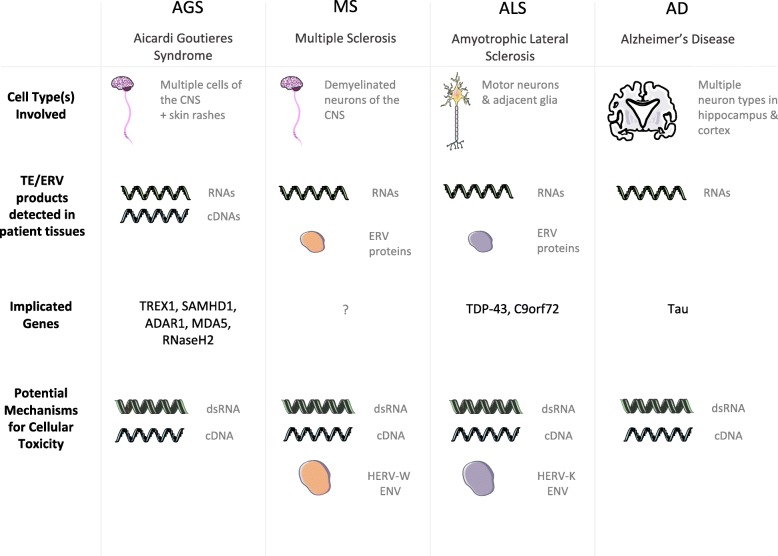
Transposable element (TE) activity in four neurological disorders: Aicardi-Goutieres Syndrome (AGS), Multiple Sclerosis (MS), Amyotrophic Lateral Sclerosis (ALS), and Alzheimer’s Disease (AD). In AGS and MS, TE nucleic acids and endogenous retroviral (ERV) proteins may be driving inflammation through innate immune sensing pathways. In ALS and AD, the pathogenic effects of TEs appear more localized to either motor neurons (in ALS), and hippocampal or cortical neurons (in AD). Innate immune pathways are activated by double-stranded RNAs and cDNAs produced by TE/ERV transcription and reverse transcription, respectively; this is the primary mechanism implicated in AGS, and could be at play in the other disorders. In addition, envelope proteins from the HERVW and HERVK class have been shown to be neurotoxic when expressed, and implicated in MS and ALS, respectively. Increased mobilization of fully competent TEs has not been convincingly demonstrated for any neurodegenerative disorder, though this mechanism has not been fully tested

In the case of neurological diseases, the best evidence for differential TE activity has come from detection of elevated TE-associated RNAs, cDNAs, and proteins in patient samples. While specific TE derived products have different consequences, the most commonly implicated pathogenic mechanisms are an inflammatory response to dsRNAs and/or cDNAs, or a direct cytotoxic response to specific proteins. The rest of this review will focus on the evidence for TE activity in four neurological disorders that have both evidence of TE products in diseased patient tissues as well as model organism support for pathogenicity downstream of TE activity. These include Aicardi-Goutieres syndrome (AGS), Multiple Sclerosis (MS), Amyotrophic Lateral Sclerosis (ALS), and Alzheimer’s Disease (AD). The diseases have been roughly divided into those that show evidence of retrotransposon induced general inflammation (AGS and MS) and those that show neurotoxic effects of retrotransposon products (ALS and AD). Figure 1 summarizes the evidence implicating retrotransposons in each of these diseases, which will be discussed in detail in the following sections. Table 1 provides a list of all named genes discussed in this review, both those that contribute to disease as well as those involved generally in retrotransposon regulation.

**Table 1:** A Glossary of all gene names cited in this review including the official symbol, common names, known function, and potential association with each of the four neurodegenerative diseases: Aicardi-Goutieres Syndrome (AGS), Amyotrophic Lateral Sclerosis (ALS), Alzheimer’s Disease (AD), and Multiple Sclerosis (MS). The name of the associated neurodegenerative disease is enclosed in parentheses if a disease-associated mutation has not been identified in the Online Mendelian Inheritance in Man (OMIM) database, but has still been implicated due to other experimental evidence discussed in this review

Gene symbol	Gene name	Synonym	Known function	Associated disorder^a^
ADAR	Adenosine Deaminase, RNA Specific		Enzyme converting adenosine to inosine by deamination	AGS
APP	Amyloid Beta Precursor Protein	A-beta	Cell surface receptor with roles in neurite growth, neuronal adhesion and axonogenesis	AD
ARC	Activity Regulated Cytoskeleton Associated Protein		Regulator of synaptic plasticity that mediate intercellular RNA transfer in the nervous system.	(AD)
ASH1L	ASH1 Like Histone Lysine Methyltransferase	ASH1	Histone methyltransferase specifically methylating “Lys-36” of histone H3	
BPTF	Bromodomain PHD Finger Transcription Factor		Histone-binding component of nucleosome-remodeling factor	
C9ORF72	C9orf72-SMCR8 Complex Subunit Chromosome 9 Open Reading Frame 72		Component of the C9orf72-SMCR8 complex that has guanine nucleotide exchange factor activity and regulates autophagy	ALS
CGAS	Cyclic GMP-AMP Synthase		Catalyzes formation of cyclic GMP-AMP (cGAMP) from ATP and GTP	
DDX58	DExD/H-Box Helicase 58	RIG-I	Cytoplasmic sensor of viral nucleic acids	
ERVW-1	Endogenous Retrovirus Group W Member 1, Envelope		Induces trophoblast fusion and formation of a placental syncytium	(MS)
ERVW-2	Endogenous Retrovirus Group W Member 2		None reported	(MS)
IFIH1	Interferon Induced With Helicase C Domain 1	MDA5	Cytoplasmic sensor of viral nucleic acids	AGS
IRF1	Interferon Regulatory Factor 1		Activator of genes involved in both innate and acquired immune responses	
PIWIL1	Piwi Like RNA-Mediated Gene Silencing 1		Endoribonuclease that represses transposable elements in postnatal germ cells	
RNASEH2A	Ribonuclease H2 Subunit A		Catalytic subunit of RNase H2 that degrades RNA of RNA:DNA hybrids	AGS
RNASEH2B	Ribonuclease H2 Subunit B		Non catalytic subunit of RNase H2	AGS
RNASEH2C	Ribonuclease H2 Subunit C		Non catalytic subunit of RNase H2	AGS
SAMHD1	SAM And HD Domain Containing Deoxynucleoside Triphosphate Triphosphohydrolase 1		Host restriction factor involved in defense response to virus	AGS
TARDBP	TAR DNA Binding Protein	TDP-43	RNA-binding protein involved in various steps of RNA biogenesis and processing	ALS
TMEM173	Transmembrane Protein 173	STING	Receptor that detects cytosolic nucleic acids	
TNF	Tumor Necrosis Factor	TNF-alpha	Pro-inflammatory cytokine	
TNFSF14	Tumor Necrosis Factor Superfamily Member 14	LIGHT	TNF superfamily ligand	
TREX1	Three Prime Repair Exonuclease 1		3′-to-5′ DNA exonuclease	AGS

^a^The associated disorder is enclosed in parentheses if the gene-disorder association is not listed in OMIM, but has been implicated due to other experimental evidence in one of the studies discussed

## Evidence for retrotransposon activity in Aicardi-Goutieres syndrome

Aicardi-Goutieres Syndrome (AGS) (OMIM 225750) is a genetic disorder caused by abnormal activation of the type I interferon pathway. The disorder typically manifests in infants within their first year of life and is characterized by general inflammation. Other clinical symptoms include severe encephalopathy with dystonia, spasticity, intermittent sterile pyrexia, basal ganglia calcifications, leukodystrophy, and a lymphocytic CSF pleocytosis [24, 33, 34].

AGS can be caused by mutations in the three prime repair exonuclease 1 (TREX1) [35], SAM and HD domain containing deoxynucleoside triphosphate triphosphohydrolase 1 (SAMHD1) [36], adenosine deaminase RNA specific (ADAR) [37], interferon induced with helicase C domain 1 (IFIH1) [38] genes, or subunits of the RNase H2 complex [39]. Intriguingly, these genes are involved in the modulation of cytosolic nucleic acid species, and pathogenic mutations lead to increased type I interferon activity that mimics an innate response against viral infection in nearly all AGS patients [40, 41]. The involvement of the aforementioned genes suggests that endogenous nucleic acid products could accumulate, and become recognized as foreign in AGS, triggering an innate immune response against the host.

Retrotransposons are implicated as a source of immunogenic endogenous nucleic acid products in AGS, though the two sub-classes implicated, L1 and Alu, appear to operate through different mechanisms. Both SAMHD1 and TREX1 alter LINE-1 activity in human cells and mouse models. Depletion of TREX1 results in the accumulation of reverse-transcribed cytosolic single-stranded DNA fragments containing L1 sequences, and causes an increase in L1 retrotransposition events in reporter assays [36, 37, 42, 43]. This is replicated by pathogenic TREX1 variants found in AGS patients [42, 44]. The exonuclease activity of TREX1 appears dispensable for repressing L1 activity, with TREX1 associating with and depleting the ORF1p protein via proteasome-mediated proteolysis [42]. L1 RNA is upregulated in TREX1-deficient astrocytes but not in neurons, and the higher reverse transcriptase activity (perhaps mediated by accumulation of single-stranded DNA fragments) activates the secretion of interferon from the astrocytes that leads to neurotoxicity [38, 43].

Other proteins implicated in AGS can regulate L1 elements. Depletion of SAMHD1 by siRNA leads to increased L1 retrotransposition in reporter assays [1–3, 45, 46], which is mimicked by mutations in the HD domain or the allosteric GTF binding region, but not from the loss of its dNTP nuclease activity [45, 47]. The subcellular localization of SAMHD1 correlates with its capacity for L1 regulation. Mutants that preferentially localize to the nucleus exhibit defective suppression [48], while expression of a cytoplasmically restricted SAMHD1 in HEK293T (through mutation of its nuclear localization signal) enhances L1 suppression. The SAMHD1 protein also associates with L1 ORF2p [47, 48] and reduces its expression, leading to lower reverse transcriptase activity [45, 48]. This is likely mediated by the formation of stress granules, which sequester L1 proteins [46]. ADAR and RNaseH2 complexes may also regulate L1 - depletion of ADAR increases L1 retrotransposition, for example, but the molecular mechanism remains unclear [49].

The role of Alu elements as immunogenic endogenous nucleic acid products in AGS differs from L1. Whereas L1 reverse transcriptase is implicated in activating the type I interferon pathway, the presence of Alu embedded in cellular transcripts appears to directly cause the inflammatory response. Genome-wide characterization of ADAR adenosine to inosine (A-to-I) editing revealed enrichment at Alu elements located at 3′ UTR or intronic regions of a cellular transcript [50]. Many of these Alu elements are in an inverted repeat configuration less than 1 kb apart on a single cellular transcript [51]. In the absence of ADAR, these Alu elements no longer show A-to-I editing, and strongly associate with the cytosolic dsRNA sensor, IFIH1 (also known as melanoma differentiation-associated gene 5 or MDA5), resulting in the activation of the interferon pathway [42, 44, 51]. IFIH1 mutations in AGS patients show increased affinity for endogenous transcripts with inverted Alu repeats, despite ADAR-mediated A-to-I editing [42, 51]. The identification of Alu inverted repeats as a possible endogenous trigger for AGS has solidified a model where ADAR serves to edit “self” RNA at regions of high secondary structures to prevent them from being recognized as foreign by IFIH1 [50–55].

The observation that reverse transcriptase activity is increased by depletion of TREX1 and SAMHD1 inspired researchers to try reverse transcriptase inhibitors (RTIs) to treat AGS. In a *Trex1* mouse model, one study showed amelioration of inflammatory myocarditis by the RTIs Truvada and Viramune [56], while another study failed to demonstrate a reduction in interferon response in the same model system [57]. Despite these conflicting results in mouse models, a pilot study of three reverse transcriptase inhibitors (abacavir, lamivudine, and zidovudine) showed a reduced interferon response in AGS patients as measured by expression of six interferon stimulated genes [58]. Though the study was small (8 patients) and open label/un-blinded, they were able to detect a reduction in interferon activity in blood and CSF samples as well as increased cerebral blood flow for all patients during the 12 months they were receiving therapy [58], suggesting this will be a promising therapeutic strategy. Further studies are required to determine if the pathogenic capacity of retrotransposons in AGS is restricted to the generation of endogenous immunogenic molecules, or might also be mediated by their retrotransposition.

Finally, in addition to the evidence for retrotransposon-mediated contributions to AGS pathophysiology, two studies demonstrated much higher rates of micronuclei formation in the Rnaseh2b mutant mouse model of AGS [59, 60]. Micronuceli are membrane-enclosed cytosolic structures containing fragments of genomic DNA not incorporated into the daughter nuclei during mitosis, arising as a consequence of DNA damage and/or aneuploidy. The amount of micronuclear DNA in the Rnaseh2b mutants correlated with cGAS and STING pathway activation, suggesting that generic DNA, and not just retrotransposon cDNA, may induce auto-inflammatory mechanisms in this AGS mouse model. Moreover, at least two studies [59] have shown that RNASEH2B appears to be required for L1 transposition, suggesting that L1 cDNA is unlikely to increase in the absence of RNASEH2B, and may not be the trigger for auto-inflammation for this particular gene mutation.

## Evidence for HERV activity in MS

Multiple sclerosis (MS) (OMIM 126200) is a chronic CNS disorder involving autoimmune-mediated demyelination. Patients most often present with focal neurological deficits (e.g. vision loss, altered/loss of sensation, motor deficits, or cognitive impairment) that localize to distinct areas (“plaques”) of demyelination in the brain, spinal cord, and/or optic nerves. The deficits may partly or fully resolve over the ensuing weeks, with later “relapses” involving new deficits in different CNS regions (“relapsing-remitting” MS), or there may be a more gradual progressive deterioration without improvement (“primary progressive MS”). Cases with a relapsing-remitting course can also later transform into a progressive course without improvement between discrete attacks (“secondary progressive MS”) [61]. Genetic association studies have identified over 200 risk loci for MS, the strongest of which lies in the major histocompatibility complex (MHC) locus [62].

There is evidence that retrotransposons, particularly human endogenous retroviruses (HERVs), may be associated with the development and/or progression of MS. Cerebrospinal fluid (CSF) from MS patients has been shown to contain viral particles and reverse transcriptase activity [63]. Subsequent studies identified retroviral-like sequences in both CSF [64, 65] and serum [66, 67] of MS patients, with high homology to the human endogenous retrovirus group W (HERVW) [68]. Immunohistochemistry of MS lesions in postmortem CNS tissues show that the envelope gene of HERVW (HERVW-Env) is upregulated both at the RNA and protein levels in activated microglia and reactive astrocytes, but not in neurons or oligodendrocytes [31, 67, 69, 70]. The HERVW-Env protein induces a pro-inflammatory response in human fetal astrocytes [31], likely through TLR4 [71], and also activates genes associated with endoplasmic reticulum stress [72]. Furthermore, soluble factors released from primary rat astrocyte cultures overexpressing HERVW-Env reduce the production of myelin in cultured oligodendrocytes [72] and eventually lead to oligodendrocyte damage and death [31]. HERVW-Env proteins have also been detected in peripheral blood mononuclear cells (PBMC) [73, 74], serum and cerebral spinal fluids [75] of MS patients. MS patients with active disease (ie, those exhibiting worsening neurological dysfunction) express more HERVW-Env proteins on the cell surface of PBMCs than healthy control subjects or MS patients in remission [74]. Indeed, the transcript levels of HERVW-Env in PBMCs, as measured by RT-PCR, correlates with disease severity [76]. In contrast to these studies, another group failed to find consistently elevated HERVW-Env transcripts in MS patient tissues [77, 78], and an additional study has raised concerns about the early qPCR assays used for HERVW transcript detection [79].

In addition to full length HERVW transposable elements, two genes derived from HERVW-Env, ERVW-1 and ERVW-2, have also been identified in the human genome, and their aberrant expression could potentially contribute to the accumulation of HERVW-Env transcripts and protein. However, these genes lack other components that are likely necessary to generate inflammatory dsRNAs and cDNAs [77, 80–83]. While they share high sequence homology to the HERVW-Env transcripts reported in MS [31, 69], evidence for pathogenicity is presently lacking.

The association between HERVW-Env protein and MS has led to the development of a monoclonal antibody (GNbAC1), currently in clinical trials as a potential therapeutic for the disease. A phase I study of 33 healthy individuals indicate that GNbAC1 is well tolerated [84]. A phase IIa study of 10 patients shows a decrease in p38 phosphorylation in monocytes (a readout of decreased TLR4 signaling) after treatment with GNbAC1 over 6 months, without adverse effects on the normal functions of the immune system [85]. Another phase IIa study also shows a reduction of HERVW transcripts in individuals after 6 months of GNbAC1 treatment compared with placebo (5 MS patients in each group) [86]. No adverse effects, such as inadvertent increase in disease activity or immunosuppression, were observed in the GNbAC1 treated individuals either at the end of the 6 month period [86], nor in the 6 months afterwards [87]. However, while subsequent results showed a potential association with remyelination in patients on GNbAC1, there was no signal of immunomodulatory effects of the treatment, raising questions as to the mode of action [88].

HERVW-Env has also been implicated in Chronic Inflammatory Demyelinating Polyradiculoneuropathy (CIDP), which causes demyelination in the peripheral nervous system. HERVW-Env is observed in nerve biopsies of CIDP patients, localizing to the myelin sheath and neurilemma [89]. In vitro studies show that overexpression of HERVW-Env induces IL6 and CXCL10 expression (typically elevated in CIDP patients) through the TLR4 receptor in primary human Schwann cell cultures [89]. These parallels to findings in MS suggest that GNbAC1 could be considered as a novel therapy in CIDP as well.

In addition to HERVW, human endogenous retrovirus group H (HERVH) has also been implicated in MS. Association studies identified linkage disequilibrium of single nucleotide polymorphisms near a HERV-Fc1 (HERVH subtype) locus on the X-chromosome in MS patients [90]. While HERVH levels have not been measured in demyelinated tissues, active MS patients exhibit a significant increase in HERV-Fc1 RNA in serum as compared to healthy control subjects, and MS patients in remission also have increased HERV-Fc1 RNA in monocytes compared to unaffected individuals [91].

In summary, there is substantial evidence to suggest an association between elevation of HERV transcripts/envelope protein and MS. However, there is still uncertainty as to whether HERV expression is a cause or consequence of the neuroinflammatory response. Although HERVW-Env induces a pro-inflammatory response in astrocytes [31], its expression is also increased in response to inflammation, leading to difficulties in establishing causality [72]. Furthermore, elevated expression of particular HERVs might lead to global increases in other retrotransposon transcripts and/or proteins, potentially acting as another mechanism of cellular damage. Given the multifactorial etiology of MS, HERVW-Env (and perhaps HERVs in general) may both contribute to the initial development of MS in some cases as well as amplifying any inflammatory responses to other initiating insults in the glial population, leading to cell non-autonomous damage in the central nervous system.

## Evidence for retrotransposon activity in ALS

Amyotrophic lateral sclerosis (ALS) (OMIM 105400) is a fatal neurodegenerative disorder that is characterized by progressive loss of upper and lower motor neurons. Patients initially present with either muscle weakness of the limbs or speech/swallowing difficulties, depending on the site of onset (limb or bulbar respectively). Paralysis progressively spreads throughout the motor system, affecting critical body functions and eventually resulting in death [92]. Genetic association studies have identified about 20 ALS-associated genes that can be collectively grouped into four main disease pathways: RNA metabolism, protein homeostasis, cytoskeletal components, and mitochondrial function [93]. These mutations are most commonly detected in the ~ 10% of ALS patients with a positive family history, though some are also found in sporadic (sALS) patients, such as the C9orf72 expansion which is detected in 5–10% of sALS. However, most ALS patients present without a family history of disease nor known ALS-associated mutations [94]. Despite the relatively low genetic heritability for this disease, nearly all ALS patients show aggregates of an RNA-binding protein, TARDBP/TDP-43 (TAR DNA binding protein), that pathologically accumulates in motor neurons of the motor cortex and spinal cord [95].

Several recent studies have implicated HERV retrotransposons in the development of ALS. Serum from ALS patients shows increased reverse transcriptase (RT) activity compared to healthy controls [96–99], though there is some evidence of elevated RT activity in the serum of ALS patient relatives [97]. Studies of RT activity in the cerebrospinal fluids of ALS patients either failed to identify a significant increase [98], or only in a very small subset of patients (1/25) [99]. Immunohistochemistry of post-mortem brains of ALS patients uncovered an increase in human endogenous virus – group K (HERVK) transcripts [100, 101], and follow-up studies showed the HERVK reverse transcriptase (HERVK-RT) protein localized within the nucleus and cytoplasmic foci of cortical pyramidal neurons, and is significantly more abundant in the prefrontal and motor cortices of ALS patients compared to unaffected individuals [100]. HERVK envelope protein (HERVK-Env) was also detected in the cytoplasm of pyramidal neurons in the cortex and in the anterior neural horn of the spinal cord in ALS patients, but not in glial cells or white matter [101]. Enzyme-linked immunosorbent assay (ELISA) also detected elevated levels of HERVK-Env peptide fragments in the sera and cerebrospinal fluids of ALS patients, compared to healthy controls and patients with other neurological disorders (e.g. Alzheimer’s and MS) [75]. The levels of HERVK-Env peptides in both sera and cerebrospinal fluids also correlated with poorer ALS Functional Rating Scale – revised score (ALSFRS-R), suggesting a potential marker for ALS disease progression [75].

In vitro and in vivo overexpression of HERVK-Env results in significant dendritic defects and neuronal cell death, with a transgenic mouse model showing reduction of corticospinal motor neurons, decreased motor cortex thickness and neuronal loss of upper and lower motor neurons, decreased motor cortex thickness, limb muscle atrophy and denervation [101]. These animals also develop progressive motor dysfunction, akin to ALS patients [101]. In transgenic Drosophila models, upregulation of transposable elements (including gypsy, an invertebrate LTR retrotransposon encoding an ERV-like envelope protein) was seen in transgenic Drosophila models that over-express the ALS-associated gene TARDBP/TDP-43. In these models, aggregation of TDP-43 protein and consequent TE upregulation lead to both neuronal and glial cell death, which could be ameliorated by knockdown of the most abundant Drosophila TE, gypsy [102].

The molecular mechanism regulating HERVK expression in ALS remains unresolved. Pro-inflammatory signals, such as TNF alpha and TNF superfamily member 14 (TNFSF14/LIGHT), have been shown to activate HERVK expression levels in vitro in neurons and astrocytes, respectively. This is likely mediated by Interferon Regulatory Factor 1 (IRF1) and NF-kappa-B signaling, which were shown to be upregulated in vitro upon addition of the aforementioned pro-inflammatory signals, and upregulated and nuclear enriched in HERVK positive pyramidal neurons in the cortex of ALS patients [103]. However, it remains unclear if HERVK expression is initiated or merely amplified by neuroinflammation. Another candidate implicated in HERVK regulation in ALS is TARDBP/TDP-43 [104]. The first hints of possible interaction between HERVK and TDP-43 in ALS was their co-localization in neurons of ALS patients [100]. Analyses of genome-wide RNA binding identified direct binding of TDP-43 to RNA containing transposable elements (including L1, Alu and ERV), and that this association was reduced in patients with dysfunctional TDP-43 protein aggregates [105]. A direct association was also shown with the transfection of TDP-43 into human neurons leading to the accumulation of HERVK transcripts and HERVK-Env protein [101]. In a related study, over-expressing human TDP-43 in Drosophila neuron and glial cells resulted in increased expression of multiple retrotransposons, with the greatest effects on the gypsy viral-like LTR retrotransposon [102]. Interestingly, overexpression of TDP-43 in Drosophila glial cells caused greater retrotransposon upregulation than in neuronal cells. Although TDP-43 overexpression in both cell types lead to motor dysfunction, the disease progression was more rapid in a glial ectopic expression model, with significant TDP-43 phosphorylation, cytoplasmic accumulation and cell death [102]. However, other studies have shown that overexpression of TDP-43 alone was insufficient to increase HERVK transcripts of fetal astrocytes or neuronal cultures in vitro, and required proteasomal deficiencies and/or inflammatory signals [106]. Intriguingly, both the overexpression and depletion of TDP-43 in mouse models have been shown to significantly upregulate transposable element expression, including that of ERVs [105]. Variant forms of TDP-43 can self-aggregate into cytoplasmic inclusions in neurons of ALS patients [107–109], and it is possible that overexpression of TDP-43, rather than increasing the functional protein level, might enhance self-aggregation and further deplete TDP-43 in the nucleus. This is consistent with experiments showing that N- or C-terminal truncated TDP-43 (known to enhance aggregation [110]) more strongly promote cytoplasmic aggregation of HERVK proteins to stress granules in astrocytes [106]. However, this is in contrast with a study demonstrating that knockdown of TDP-43 reduces HERVK expression [101], as TDP-43 was shown to bind to the HERVK LTR [101, 106] and enhance Pol-II association [101]. Additional research is needed to determine if accumulation or depletion of TDP-43 (or both) mediates up-regulation of HERVK and other retrotransposons. As for other retrotransposons, at least one study has shown that depletion of nuclear TDP-43 is associated with increased L1HS accumulation in ALS post-mortem tissue, and that loss of functional TDP-43 from human cells led to an increase in L1HS retrotransposition activity [111].

TDP-43 may not be the only link between ALS disease and retrotransposon expression. Several studies have suggested a correlation between increased retrotransposon expression levels and hexanucleotide (GGGGCC) repeat expansion mutations in the non-coding region of C9orf72 [112–114]. C9orf72 is the most commonly mutated gene in familial forms of ALS, as well as a subset of sporadic ALS disease [113–117]. Transcriptome profiling studies showed that transposable element expression correlated more strongly with the presence C9orf72 repeat expansion in ALS patients, as compared to TDP-43 transcript level or phosphorylated TDP-43 protein levels [112, 115–117]. In these studies, the link between C9orf72 and retrotransposon expression was indirect, with evidence showing that C9orf72 peptides displaced one of the major heterochromatin proteins (HP1), resulting in a relaxation of heterochromatin structures and accumulation of dsRNAs from heterochromatic retrotransposons [112, 114].

In contrast to the multiple studies showing increased TE and ERV products in ALS patients tissues and animal models of disease, three recent studies have failed to find elevated levels of HERVK transcripts in ALS patient tissues [113, 114, 118, 119]. Two studies argue that HERVK transcripts show no difference between ALS patients and unaffected individuals [113, 118, 119], one found no detectable HERVK-Env protein in cortex and spinal cord by Western analysis [118, 119], while the third found no evidence for general retrotransposon elevation after reanalyzing published datasets [113, 118]. This may be due to differences in methodology, but could also suggest heterogeneity in retrotransposon levels among ALS patients. As to prevalence in ALS populations, recent reviews summarizing the function of ALS-associated genes has led to a growing appreciation that ALS may be a molecularly heterogeneous disease, with multiple parallel pathways leading towards a similar phenotypic and clinical outcome [94, 113]. This could explain the conflicting observations in ALS patient samples regarding HERVK expression and its correlation with TDP-43 expression or pathology in smaller subsets of sporadic patients. In contrast, patients carrying C9orf72 mutations might represent a more similar patient group and show more consistency in terms of molecular pathways altered. It is therefore highly probable that ALS patients represent a mixture of distinct molecular subtypes that show distinguishable differences in retrotransposon expression and/or alterations in multiple molecular pathways. Thus, it is important to characterize large ALS cohorts to definitively establish the potential role and impact of retrotransposon activity in the etiology of the disease.

## Evidence for heterochromatin relaxation in Alzheimer’s disease

Alzheimer’s disease (AD, OMIM 104300) is a neurodegenerative disorder that is marked by progressive damage and loss of neurons in the central nervous system. It is characterized pathologically by an accumulation of intracellular neurofibrillary tangles of Tau protein and extracellular amyloid plaques in the affected brain regions. Patients most commonly present with memory and language issues, later exhibiting decline in general cognitive function and control of body functions, ultimately leading to death [94, 120]. Genetic association studies over the years have implicated more than 20 risk alleles for dysfunctional amyloid processing, lipid metabolism, immune response, and general synaptic function [120, 121]. However, these genes do not explain all of the estimated heritability of AD, and disease onset is likely to involve a complex interplay between genetic and environmental factors [121, 122].

The role of retrotransposons in Alzheimer’s disease is not well defined, but there is evidence that the epigenetic landscape induced by Tau pathology could allow for general transposon re-activation. Specifically, retrotransposons have the highest density among the heterochromatic regions that are normally transcriptionally silent. Overexpression of Tau in Drosophila shows significant loss of heterochromatin across the genome, upregulation of Ago3 (the Drosophila homolog of PIWIL1), and significant locomotor dysfunction. The brains of Alzheimer’s patients similarly show diffuse H3K9 di-methylation and altered distributions of the major heterochromatin protein HP1 in pyramidal neurons positive for disease-associated Tau, as well as upregulation of PIWIL1 [122, 123]. Overexpression of Tau in aging Drosophila brains also increased expression of certain retrotransposons [123, 124], and knockdown of a heterochromatin-associated gene, BPTF, enhanced the locomotor dysfunction of the transgenic Tau-overexpressing Drosophila, while knockout of the ASH1L histone lysine methyltransferase (euchromatin-associated) attenuated the phenotype [123, 124]. This suggests that the pervasive euchromatin state induced by Tau overexpression could be modified through targeting of epigenetic regulators, and might be a possible avenue for treatment.

In addition to chromatin changes, analysis of Alzheimer’s brain samples also revealed gene expression profiles that resemble fetal brain, with expression of several pluripotency-associated factors [123]. This suggests the possibility for increased L1 retrotransposition, which has previously been reported in neural progenitor cells of fetal brains [11, 123, 125], especially given the observation that retrotransposon expression shows positive association with Tau pathology [11, 124, 125]. While there are some indications of novel retrotransposition events in Alzheimer’s patients [124, 126] and Drosophila models of Tau pathology [124, 126], their extent and contribution to pathology remains unresolved. Along with observations of higher L1 methylation [124, 127] and no detectable differences in the number of “active” L1 copies [126, 127] in Alzheimer’s patients compared to unaffected individuals, there are still open questions as to whether L1HS specifically (among all retrotransposons) plays a role in the etiology of Alzheimer’s disease.

Endogenous retrovirus levels have been shown to positively correlate with Tau pathology in postmortem dorsolateral prefrontal cortex of individuals from the Religious Orders Study and Rush Memory and Aging Project (ROSMAP) project [124, 126]. Overexpression of Tau in aging Drosophila brain caused an increase in LTR-class retrotransposons, while Alzheimer’s patients with severe neurofibrillary tangles show enrichment of H3K9 acetylation marks around HERV-Fc1 loci [124]. Yet, there have been no reports of increased reverse transcriptase activity or presence of envelope proteins in Alzheimer’s patients or animal models thus far.

Mutations and duplications of amyloid beta precursor protein (APP) gene have been implicated in familial Alzheimer’s disease [124, 128–130], while brains of sporadic Alzheimer’s patients show increased mosaic APP copy number variation compared to healthy individuals [128–131]. A recent study detected novel genomic copies of APP enriched in neurons of sporadic Alzheimer’s patients that are reminiscent of processed/retroposed pseudogenes [131, 132]. These novel APP copies lack intronic sequence, and often contain intra-exonic junctions (partial exon fusion) that ablate central exons of the APP gene. Ectopic expression of human full-length APP in mouse brains show that the generation of novel APP genomic copies is transcription-dependent [132]. While the study suggests that APP could be undergoing somatic retro-insertion (which can be mediated by retrotransposons such as L1), it remains unclear if these novel APP variants have a pathogenic role in Alzheimer’s disease, or if they are a by-product of other underlying pathogenic mechanisms. While these novel APP variants are enriched in neurons of Alzheimer’s patients, it is unclear if this is due to a chronic/ongoing elevation of retrotransposition activity in these patients (of which there is no evidence thus far), or from an elevated spike of retro-insertion at an unspecified point during the patients’ life. Additional studies are required to address these questions.

Finally, there exists within the genome several host genes derived from endogenous retrotransposon sequences that might provide a more distant link between TEs/ERVs and neurodegenerative disease. ARC is a neuronal gene involved in trafficking of glutamate receptors at the synapse [132–135]. It associates with PSEN1 and mediates the internalization of APP from post mitotic dendrites [133–136]. The ARC protein sequence has high homology to retroviral Gag proteins [136, 137], and has been shown to assemble into a viral-like capsid that encapsulates RNA for intercellular transport [137–139]. ARC has been previously implicated in Alzheimer’s disease as an enhancer of A-beta production, with increased ARC protein in the medial frontal cortex of Alzheimer’s patients. Arc enhances the association of gamma secretase with APP in the endosome, and Alzheimer’s mouse models lacking Arc show reduced plaque and A-beta levels compared to those with functional Arc [136, 138, 139]. However, an intriguing observation in Drosophila found that Arc capsids could occasionally encapsulate endogenous retroviral RNA [136, 138]. This raises an interesting prospect that ARC could mediate the spread of endogenous retroviral sequences between neurons in neurodegenerative disorders (e.g. from cells with elevated HERV expression). As such, Arc would likely provide a transport system to allow for the spread of ERV RNAs between cells, rather than a factor that induces elevated expression of ERVs.

## Conclusions

In summary, there is ample evidence for elevation of certain retrotransposon RNAs and protein products in postmortem patient tissues for multiple neurodegenerative diseases and increased RT in patient biofluids. Specific HERV Envelope proteins appear particularly neurotoxic. However, questions remain regarding 1) which particular retrotransposon products are elevated in each disease and tissue context, 2) whether these elevated levels are expected to be present in all patients with the disease or in subsets of patients, and 3) whether this elevated expression is just a marker of cellular dysfunction in each disease or is pathogenic. Studies in Aicardi Goutieres Syndrome (AGS) showed the best evidence to date for elevated retrotransposon transcripts being present in patient tissues and causing neuroinflammation through aberrant activation of innate immune complexes. In AGS, patients carried genetic mutations in complexes that normally process endogenous retroelements, providing a mechanistic explanation for elevated retrotransposon levels. Studies in ALS and MS have predominantly focused on the potential neurotoxicity of HERV Envelope proteins from HERVK and HERVW, respectively, though inflammatory responses to transcripts could also play a role, especially given the induction of inflammatory pathways seen in both diseases. Finally, studies in Alzheimer’s disease suggest an indirect elevation of retrotransposon levels through heterochromatin relaxation induced by Tau pathology. Chromatin relaxation is thought to cause a widespread depletion of heterochromatin associated proteins from normally closed genomic regions with a consequent opening of heterochromatin and increase of passive transcription from these regions. This process has similarly been hypothesized as a mechanism for inducing retrotransposon expression in the subset of ALS patients carrying C9orf72 mutations [114, 138]. Studies focused on normally aging tissues from rodent models have shown that retrotransposon de-silencing may happen as a consequence of normal age-related alterations in chromatin state [27, 28, 114, 140, 141], and that inflammatory pathways are induced downstream of retrotransposon activation. These studies suggest that elevated basal retrotransposon levels may be a general feature of aging that makes retrotransposon induced stress more likely in aging-related neurodegenerative diseases.

One aging-related neurodegenerative disorder not discussed above, but potentially related, affects neurons in the frontal and temporal lobes, Frontotemporal Dementia (FTD). Specifically, a subset of ALS patients also develop cognitive issues or behavioral changes that are understood to result from FTD, an umbrella term for a group of clinical dementia syndromes that correlate with the pathologic finding of Frontotemporal Lobar Degeneration (FTLD) (OMIM 600274). The ALS-Frontotemporal Spectrum Disorder is more common in patients with familial forms of ALS. Mutations in the C9orf72 gene are the most common cause of hereditary FTD, ALS, and ALS with FTD. Several other genes are now recognized to cause both diseases. This has led to speculation that the familial forms of ALS and some forms of FTLD might be related genetic diseases that predominantly differ in terms of the affected tissues at onset, but may share molecular mechanisms of pathogenesis [27, 28, 140–145]. FTD and its spectrum disorders were not included above due to a current lack of direct evidence linking TEs to FTD in patient tissues, though any discussion of TE expression downstream of TDP-43 and C9orf72 induced pathology could apply to some FTD-spectrum disorders as well.

None of these studies have yet shown clear evidence for de novo insertions, or “hopping,” of the retrotransposons in decedent patient tissues or laboratory models, though it is possible that improved methods for identifying somatic de novo insertions may shed more light on this possibility. As sequencing technologies improve with longer reads [142–147], better protocols for transposon insertion profiling [146–151], and better computational tools to handle repetitive genomic regions [148–152], it may be easier to detect retrotransposon products ranging from specific loci generating elevated retrotransposon transcripts to polymorphic and de novo genome insertions.

## Data Availability

Not applicable.

## Abbreviations

A to I: Adenosine to inosine
AD: Alzheimer’s disease
AGS: Aicardi-Goutieres syndrome
ALS: Amyotrophic lateral sclerosis
ALSFRS-R: Amyotrophic lateral sclerosis functional rating scale - revised
cDNA: Complementary deoxyribonucleic acids
CIDP: Chronic Inflammatory Demyelinating Polyradiculoneuropathy
CNS: Central nervous system
CSF: Cerebrospinal fluid
DNA: Deoxyribonucleic acids
dNTP: Deoxyribonucleoside Tri-phosphate
dsRNA: Double-stranded Ribonucleic acids
ELISA: Enzyme-linked immunosorbent assay
ERV: Endogenous retrovirus
FTD: Frontotemporal dementia
FTLD: Frontotemporal lobar degeneration
GTF: Guanosine Tri-phosphate
H3K9: Histone 3, Lysine 9
HD domain: Histidine/Aspartate-rich domain
HEK293T: Human embryonic kidney 293 cells with SV40 large T antigen
HERV: Human(−specific) endogenous retrovirus
HERVH: Human(−specific) endogenous retrovirus, group H
HERVK: Human(−specific) endogenous retrovirus, group K
HERVK-Env: Human(−specific) endogenous retrovirus, group K, envelope
HERVK-RT: Human(−specific) endogenous retrovirus, group K, reverse transcriptase
HERVW: Human(−specific) endogenous retrovirus, group W
HERVW-Env: Human(−specific) endogenous retrovirus, group W, envelope
L1: Long interspersed nuclear element 1
L1HS: Long interspersed nuclear element 1, *Homo sapiens*
LINE: Long interspersed nuclear element
LTR: Long terminal repeat
MHC: Major histocompatibility complex
MS: Multiple sclerosis
PBMC: Peripheral blood mononuclear cells
qPCR: Quantitative polymerase chain reaction
RNA: Ribonucleic acids
ROSMAP: Religious Orders Study and Rush Memory and Aging Project
RT: Reverse transcriptase
RTI: Reverse transcriptase inhibitor
RT-PCR: Reverse transcription and polymerase chain reaction
sALS: Sporadic amyotrophic lateral sclerosis
SAM: Sterile alpha motif
SINE: Short interspersed nuclear element
siRNA: Short interfering ribonucleic acids
SVA: SINE/VNTR/Alu elements
TE: Transposable elements
UTR: Untranslated Region
